# Supplementary material for: The interactome of intact mitochondria by cross-linking mass spectrometry provides evidence for coexisting respiratory supercomplexes
Source: Mol Cell Proteomics. 2017 Dec 8;17(2):216–32. doi: 10.1074/mcp.RA117.000470 (PMC5795388; doi:10.1074/mcp.RA117.000470)
Supplement: Supplemental Data [file supp_RA117.000470_133922_0_supp_23978_fzffwf.zip › spectra_annotation/mito_spectra_annotation/366-1-6-1-10-1.pdf]

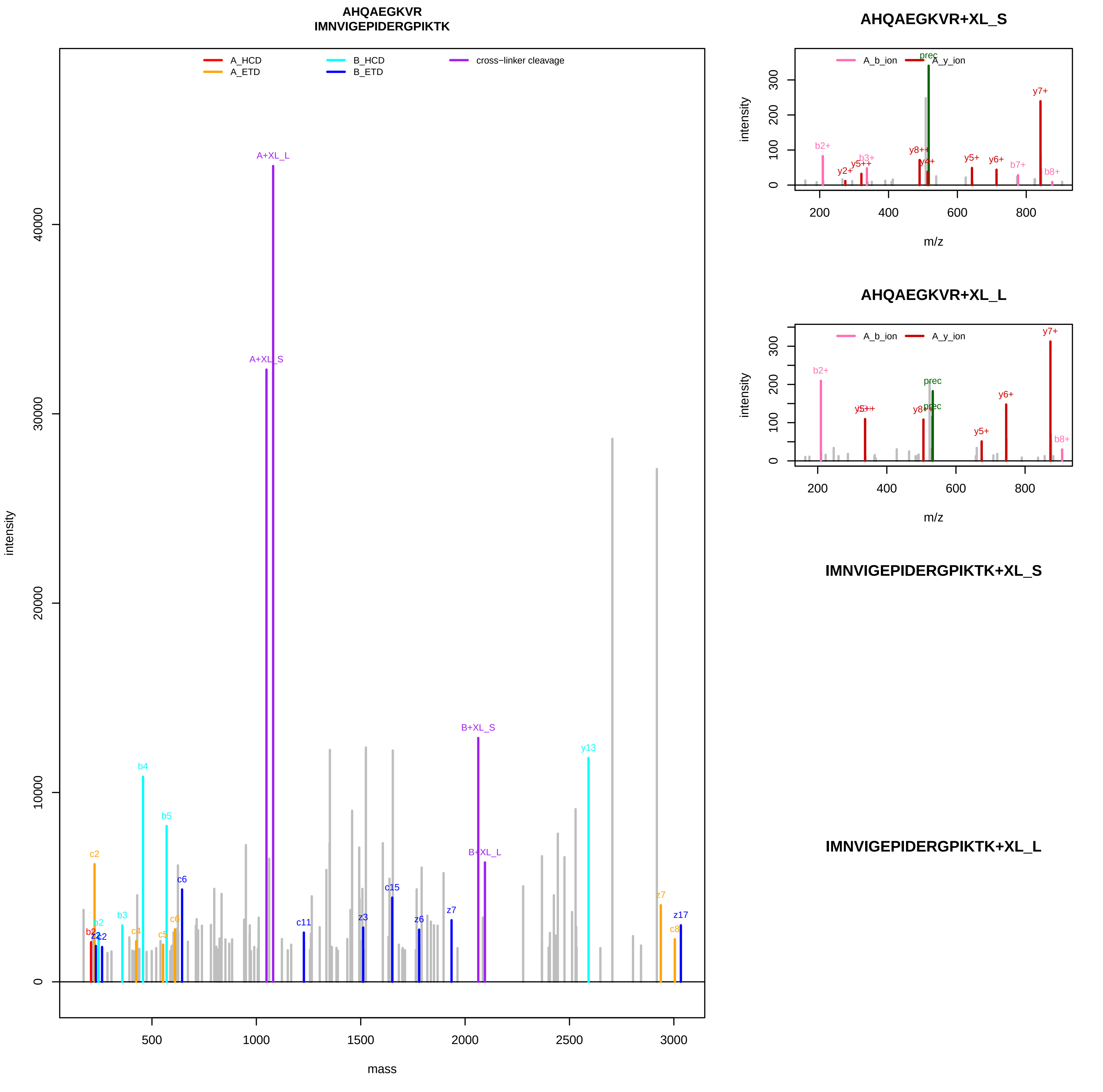
AHQAEGKVR
IMNVIGEPIDERGPIKTK
A_HCD
A_ETD
B_HCD
B_ETD
cross−linker cleavage
intensity
mass
A+XL_L
A+XL_S
B+XL_S
B+XL_L
b2
z2
b2
c2
c2
b3
b4
b5
c4
c5
c6
c6
c11
z3
c15
z6
z7
y13
z7
c8
z17
AHQAEGKVR+XL_S
A_b_ion
A_y_ion
prec
y7+
b2+
y2+
y5++
b3+
y8++
y4+
y5+
y6+
b7+
b8+
m/z
AHQAEGKVR+XL_L
y3++
prec
IMNVIGEPIDERGPIKTK+XL_S
IMNVIGEPIDERGPIKTK+XL_L
